# Melatonin Treatment Improves Insulin Resistance and Pigmentation in Obese Patients with Acanthosis Nigricans

**DOI:** 10.1155/2018/2304746

**Published:** 2018-03-12

**Authors:** Hang Sun, Xingchun Wang, Jiaqi Chen, Aaron M. Gusdon, Kexiu Song, Liang Li, Shen Qu

**Affiliations:** ^1^Department of Endocrinology and Metabolism, Shanghai Tenth People's Hospital, School of Medicine, Tongji University, Shanghai 200072, China; ^2^Department of Endocrinology and Metabolism, SuZhou Municipal Hospital, Nanjing Medical University, Nanjing, Jiangsu 210029, China; ^3^Division of Neuropathology, Department of Pathology, University of Pittsburgh School of Medicine, Pittsburgh, PA 15261, USA; ^4^Department of Neurology, Weill Cornell Medical College, New York, NY 10065, USA

## Abstract

**Objective:**

This study aimed to determine the effects of melatonin on insulin resistance in obese patients with acanthosis nigricans (AN).

**Methods:**

A total of 17 obese patients with acanthosis nigricans were recruited in a 12-week pilot open trial. Insulin sensitivity, glucose metabolism, inflammatory factors, and other biochemical parameters before and after the administration of melatonin were measured.

**Results:**

After 12 weeks of treatment with melatonin (3 mg/day), homeostasis model assessment insulin resistance index (HOMA-IR) (8.99 ± 5.10 versus 7.77 ± 5.21, *p* < 0.05) and fasting insulin (37.09 5 ± 20.26 *μ*U/ml versus 32.10 ± 20.29 *μ*U/ml, *p* < 0.05) were significantly decreased. Matsuda index (2.82 ± 1.54 versus 3.74 ± 2.02, *p* < 0.05) was significantly increased. There were also statistically significant declines in the AN scores of the neck and axilla, body weight, body mass index, body fat, visceral index, neck circumference, waist circumference, and inflammatory markers.

**Conclusions:**

It was concluded that melatonin could improve cutaneous symptoms in obese patients with acanthosis nigricans by improving insulin sensitivity and inflammatory status. This trial is registered with ClinicalTrials.gov NCT02604095.

## 1. Introduction

Melatonin is a hormone secreted by the pineal gland. The synthesis and secretion of melatonin are regulated by light intensity [1]. Investigations found that melatonin has multiple effects and acts as an antioxidant [2, 3], has anti-inflammatory properties [4, 5], regulates circadian rhythms [6], regulates immunity [7] and has antineoplastic effects [8]. Research also found that melatonin can regulate lipid metabolism [9], increase insulin sensitivity [10], regulate glucose metabolism [11], and reduce body weight [12].

Acanthosis nigricans (AN) is a disease characterized by skin pigmentation, hyperkeratosis, and velvet hyperplasia. Increased pigmentation commonly occurs in the posterior neck, axilla, and groin, and it can also be seen on the cubital fossa, labia, face, and other locations throughout the body [13]. AN can be divided into two categories: benign and malignant [14]. Studies have found that benign AN is closely related to hyperinsulinemia, insulin resistance (IR), and obesity [15, 16]. Many different topical and oral treatments have been attempted for AN. Studies reported that treatment with both metformin and rosiglitazone are useful in AN characterized by IR [17, 18]. Low-calorie diet, increasing physical activity, and weight reduction also can relieve symptoms of AN by improving the IR [19]. However, no safe and effective therapy exists especially when considering young patients with euglycemia. Patients with AN also are at increased risk of developing metabolic disorders such as diabetes and dyslipidemia.

Melatonin can regulate internal biological clocks and energy metabolism [20]. Melatonin influences insulin secretion mediated by Gi-protein-coupled melatonin receptors MT1 and MT2 [21]. It was found that melatonin treatment for one year could reduce fat mass and increase lean mass in postmenopausal women [22]. So this study was designed to determine whether treatment with melatonin is an effective treatment for AN as well as insulin resistance and metabolic disturbances.

## 2. Materials and Methods

### 2.1. Study Population

A total of 17 patients (6 males and 11 females, age 27.35 ± 6.50) were enrolled in the 12-week pilot open trial with no placebo group included. The study was approved by the ethical committee of the Shanghai Tenth People's Hospital, Tongji University, Shanghai, China, and registered by ClinicalTrials.gov (NCT02604095). Inclusion criteria included (1) aging from 18 to 60 years old, (2) having acanthosis nigricans, and (3) body mass index (BMI) exceeding 28 kg/m^2^. Exclusion criteria included (1) malignant acanthosis nigricans; (2) serious renal, adrenal, or hepatic dysfunction; (3) taking medications chronically for systemic illness; (4) lactation; (5) pregnancy; (6) active cancer; (7) taking weight-loss drugs; and (8) history of stomach reduction surgery. Each patient gave written informed voluntary consent before participating in the study.

### 2.2. Study Design

After the initial screening, age, gender, and height of each participant were recorded. Weight, BMI, and visceral index were measured with light clothes and without shoes using an Omron HBF-358 (Q40102010L01322F, Japan), with the same controlling of water, food, diuretics, alcohol and coffee intake, and urination. Neck circumference (NC), waist circumference (WC), hip circumference (HC), blood pressure (BP), and heart rate (HR) were measured twice, and the average was used for analysis.

Scores of AN were calculated as described previously [23]. There are four degrees from 0 to 4 on the AN scales, which are strongly associated with fasting insulin and BMI.

After overnight fasting, serum glucose, insulin, C-peptide (C-p), lipid profiles, free fatty acids (FFA), uric acid (UA), inflammation factors (c-reactive protein (CRP), tumor necrosis factor alpha (TNF-*α*), interleukin 6 (IL-6), and interleukin 8 (IL-8)), superoxide dismutase (SOD), alanine transaminase (ALT), aspartate transaminase (AST), creatinine (Cr), urea nitrogen (UN), free triiodothyronine (fT3), free thyroxine (fT4), and thyroid stimulating hormone (TSH) were measured. Participant also underwent a standard 75 g oral glucose-tolerance test (OGTT) after fasting overnight. Samples were collected at 0, 30, 60, 90, and 120 min, and glucose, insulin, and C-p were measured. All these measurements were performed before and after 12 weeks of treatment.

Each patient received 3 mg/day melatonin (Schiff Nutrition Group: each tablet contains melatonin 3 mg, vitamin B6 5 mg, and calcium 52 mg) given orally at bedtime for 12 weeks.

### 2.3. Statistics and Calculations

All data were analyzed using SPSS 21.0 software (Chicago, IL). The data are presented as the means ± SD or medians (interquartile range) for skewed variables or proportions for categorical variables. Student's *t*-tests were used to compare the difference between measured variables. *p* values < 0.05 were considered statistically significant. Insulin resistance and sensitivity were assessed by the homeostasis model assessment insulin resistance index (HOMA-IR; fasting insulin × fasting glucose/22.5) [24] and Matsuda index {1000/(Glu0 × Ins0 × mean-glucose × mean-insulin)^1/2^} [25].

## 3. Results

### 3.1. Insulin Resistance and Sensitivity

As shown in Table 1, melatonin treatment significantly decreased the HOMA-IR (8.99 ± 5.1047 versus 7.77 ± 5.2169, *p* < 0.05) and fasting insulin (37.0935 ± 20.26215 versus 32.1018 ± 20.29752 *μ*U/ml, *p* < 0.05) levels and induced the Matsuda index (2.82 ± 1.54 versus 3.74 ± 2.02, *p* < 0.05). There was no statistically significant change in fasting glucose and fasting C peptide levels.

### 3.2. Physiological Measures

**Figure 1:**
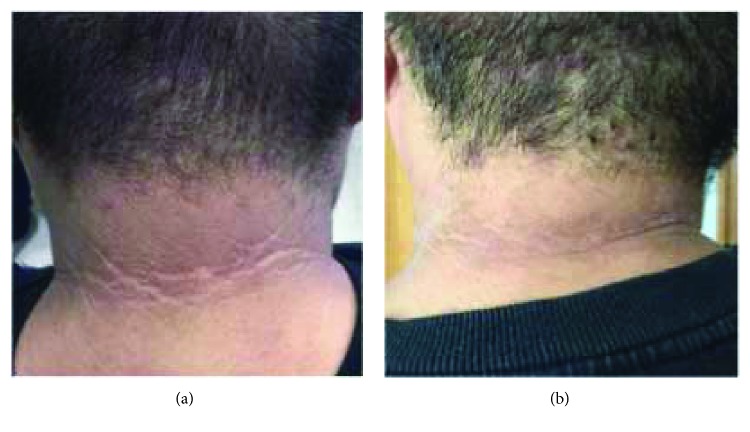
Effects of melatonin on acanthosis of neck: a typical case. (a) Baseline. (b) After 12 w treatment.

After 12 weeks of treatment with melatonin, there were statistically significant changes in the AN scores of the neck (3.35 ± 0.862 versus 2.59 ± 0.712, *p* < 0.01) and axilla (3.53 ± 0.717 versus 2.65 ± 0.702, *p* < 0.01) (Table 2). There were also significant reductions in BMI (35.741 ± 4.7844 versus 34.524 ± 4.7278 kg/m^2^, *p* < 0.01), body weight (100.912 ± 22.9884 versus 97.453 ± 21.8012 kg, *p* < 0.01), body fat (37.206 ± 5.3743% versus 35.488 ± 6.6078%, *p* < 0.05), and visceral index (17.41 ± 6.510 versus 15.82 ± 6.085, *p* < 0.01) (Table 2). Pigmentation in patients with AN was improved after treatment (Figure 1). Melatonin also decreased NC (40.718 ± 4.1652 versus 40.118 ± 4.1213 cm, *p* < 0.05), WC (111.471 ± 13.7184 versus 107.853 ± 14.2607 cm, *p* < 0.01), and HC (114.818 ± 10.6647 versus 113.488 ± 10.5441 cm, *p* < 0.05) (Table 2).

### 3.3. Lipid and Inflammatory Parameters

As shown in Table 1, there were no significant decreases in serum lipids (TC, TG, LDL-C, and HDL-C). CRP (4.6159 ± 3.27755 versus 3.6429 ± 2.89784 mg/l, *p* < 0.05) and TNF-*α* (28.0594 ± 25.81318 versus 16.9288 ± 14.92093, *p* < 0.05) were decreased significantly, but IL-6 and IL-8 did not decrease significantly. There were also no changes in serum SOD, ALT, AST, Cr, UN, fT3, fT4, and TSH (Table 2). The baseline of participants is showed in Table 3.

## 4. Discussion

In 2000, AN was established as a formal risk factor for the development of diabetes in children by the American Diabetes Association [16]. Insulin resistance commonly occurs in association with AN.

The cutaneous manifestations of AN are caused by hyperinsulinemia and the stimulation of keratinocytes and dermal fibroblasts by growth factors [26, 27]. Patients with hyperinsulinemia have increased IGF-1 levels [28]. Hyperinsulinemia is able to reduce IGF binding protein- (IGFBP-) 1 and IGFBP-2, which regulate levels of IGFs. Reduced IGFBP-1 and IGFBP-2 levels could increase free IGF-1, thereby promoting the development of papillomatosis and hyperkeratosis observed in AN [29]. There are two membrane receptors of melatonin called MT1 and MT2, which are G-protein-coupled receptors, in the periphery [30]. Both receptors are expressed in the islets of Langerhans and are involved in the regulation of glucagon secretion from *α*-cells and insulin secretion from *β*-cells [10]. Numerous studies support that activation of MT1 or MT2 leads to a reduction in second messenger 3′,5′-cyclic adenosine monophosphate (cAMP) or 3′,5′-cyclic guano-sine monophosphate (cGMP) accompanied by reduced insulin secretion [31, 32]. Picinato et al. [33] found the effects of melatonin on the phosphorylation of IGF and insulin receptors triggering their signalization cascade. Therefore, the effect of melatonin on insulin secretion and IGF may explain the mechanism of action behind the utility of treating AN with melatonin.

Most patients with AN also have severe obesity. Accumulating evidence shows that obesity is a clinical indicator for the risk of cardiovascular diseases, diabetes mellitus, and the associated metabolic syndrome [34, 35]. Somewhat surprisingly, we found that treatment with melatonin for only 12 weeks significantly decreased body weight, BMI, body fat, and visceral index of obese participants. Previous studies have shown that NC is associated with the metabolic disorders related to insulin resistance, cardiovascular diseases, metabolic syndrome, and obstructive sleep apnea syndrome [36, 37]. Melatonin was also found to decrease NC and WC, which are commonly considered as risk factors for metabolic syndrome.

Numerous studies have demonstrated that melatonin has antioxidant [2, 3] and anti-inflammatory [4] functions. In our study, serum CRP and TNF-*α* were decreased. Some investigators have suggested that the inflammation induced by metabolic surplus is different from classical inflammation [38]. TNF-*α* was the first inflammatory marker discovered in adipose tissue of obese mice [39]. As another inflammatory marker, CRP has been shown to be associated with an increased risk for development of type 2 diabetes mellitus (T2DM) [40]. We consider the anti-inflammatory function of melatonin as one of the possible mechanisms by which it improves AN.

This study had several limitations. Firstly, recruitment to diabetes trials has long been associated with placebo-mediated improvements in glycemic control and associated metabolic parameters [41]. But because of the lack resources to perform additional placebo arm studies, we do not have enough ability to recruit placebo-controlled subjects. Secondly, the sample size of the present study was relatively small. Because of the individual difference, we could not see significant changes in lipid metabolism and antioxidation functions. Secondly, we could not measure the overnight urinary 6-sulfatoxymelatonin excretion (UME), which can reflect the level of internal melatonin, of the outpatients. Further studies are necessary to prove these conclusions.

## 5. Conclusion

In summary, treatment with melatonin can decrease weight, adipose tissues, and cutaneous symptoms in obese patients with AN. It may act through improvement of insulin resistance and decreasing inflammation. However, further work must be done to elucidate its complete mechanism of action.

## Figures and Tables

**Table 1:** Effects of melatonin on various biochemical indices.

Parameters	Baseline	After 12 weeks of treatment	*p* value
HOMA-IR	8.99 ± 5.1047	7.77 ± 5.2169	0.027^∗^
Matsuda index	2.822 ± 1.5423	3.744 ± 2.0238	0.017^∗^
FPG, mmol/l	5.365 ± 0.8645	5.341 ± 0.8307	0.839
Glucose 30, mmol/l	9.853 ± 1.9872	9.259 ± 1.8416	0.044^∗^
Glucose 60, mmol/l	10.794 ± 2.8203	10.247 ± 2.7405	0.156
Glucose 120, mmol/l	8.341 ± 3.1071	8.071 ± 3.2299	0.340
Glucose 180, mmol/l	5.835 ± 2.4392	5.812 ± 1.6605	0.939
Fasting insulin, *μ*U/ml	37.0935 ± 20.26215	32.1018 ± 20.29752	0.010^∗^
Insulin 30, *μ*U/ml	149.8935 ± 54.80039	116.8088 ± 54.59683	<0.01^∗^
Insulin 60, *μ*U/ml	170.45 ± 83.12034	143.9794 ± 63.21096	0.006
Insulin 120, *μ*U/ml	160.4506 ± 85.95451	135.0529 ± 92.32970	0.018
Insulin180, *μ*U/ml	80.2476 ± 64.86455	73.1259 ± 67.18857	0.416
Fasting CP, ng/ml	4.8776 ± 1.5665	4.5759 ± 1.4895	0.130
TC, mmol/l	4.9312 ± 0.7684	4.7671 ± 0.7834	0.212
TG, mmol/l	1.9094 ± 0.7576	1.7824 ± 0.8641	0.338
HDL-C, mmol/l	1.1029 ± 0.2911	1.0741 ± 0.2430	0.422
LDL-C, mmol/l	3.0282 ± 0.6262	2.9218 ± 0.4897	0.221
FFA, mmol/l	0.588 ± 0.2233	0.506 ± 0.1600	0.084
UA, *μ*mol/l	443.518 ± 132.5464	432.635 ± 111.2079	0.400
CRP, mg/l	4.6159 ± 3.27755	3.6429 ± 2.89784	0.014^∗^
IL-6, pg/ml	15.3318 ± 13.84451	13.8276 ± 10.09683	0.527
IL-8, pg/ml	221.1482 ± 321.43527	177.6976 ± 241.08857	0.328
TNF-*α*, pg/ml	28.0594 ± 25.81318	16.9288 ± 14.92093	0.040^∗^
SOD, U/ml	154.06 ± 15.262	173.35 ± 55.623	0.166
ALT, U/l	47.224 ± 23.7542	42.929 ± 25.7068	0.391
AST, U/l	32.400 ± 16.5831	30.918 ± 13.5636	0.625
Tbil, *μ*mol/l	10.376 ± 5.3582	10.476 ± 5.2538	0.903
ALP, U/l	77.347 ± 38.9236	73.018 ± 37.5913	0.217
Cr, *μ*mol/l	59.641 ± 14.6798	60.959 ± 14.6248	0.312
UN, mmol/l	4.647 ± 1.1906	4.924 ± 1.3796	0.118
fT3, pmol/l	5.1976 ± 0.50209	5.0829 ± 0.55324	0.203
fT4, pmol/l	15.3112 ± 1.32976	15.0959 ± 1.85283	0.515
TSH, pmol/l	2.75288 ± 1.389337	2.43759 ± 1.174457	0.092

^∗^ *p* < 0.05.

**Table 2:** Physiological measures.

Parameters	Baseline	After 12 weeks of treatment	*p* value
SBP, mmHg	134.18 ± 10.690	131.88 ± 10.787	0.085
DBP, mmHg	87.53 ± 10.967	87.00 ± 11.292	0.478
HR, bmp	85.35 ± 7.533	82.35 ± 9.096	0.174
Weight, kg	100.912 ± 22.9884	97.453 ± 21.8012	0.001^∗^
Body fat, %	37.206 ± 5.3743	35.488 ± 6.6078	0.038^∗^
BMI, kg/m^2^	35.741 ± 4.7844	34.524 ± 4.7278	0.001^∗^
Visceral index	17.41 ± 6.510	15.82 ± 6.085	0.001^∗^
NC, cm	40.718 ± 4.1652	40.118 ± 4.1213	0.045^∗^
WC, cm	111.471 ± 13.7184	107.853 ± 14.2607	0.002^∗^
HC, cm	114.818 ± 10.6647	113.488 ± 10.5441	0.028^∗^
AN score, neck	3.35 ± 0.862	2.59 ± 0.712	<0.01^∗^
AN score, axilla	3.53 ± 0.717	2.65 ± 0.702	<0.01^∗^

^∗^ *p* < 0.05.

**(a):** 

Number	Sex (1: male; 2: female)	Age (year)	SBP (mmHg)	DBP (mmHg)	HR (bpm)	Height (cm)	Weight (kg)
1	1	18	134	78	89	182	131.4
2	2	19	111	66	69	154	66.4
3	2	37	126	91	103	156	69
4	2	30	125	90	84	158	86.7
5	1	29	146	110	86	166	86.2
6	2	19	130	89	78	152	73.7
7	2	39	142	94	86	155.5	88.6
8	1	34	145	95	82	172	119.8
9	2	29	128	80	89	160	93.9
10	1	28	120	90	89	182	127.6
11	2	24	132	86	92	174	100.2
12	2	25	138	86	78	167	121
13	2	28	135	79	86	165	93.8
14	2	35	136	78	87	160	99.1
15	2	22	149	94	94	178	104.6
16	1	18	132	76	83	175	103.5
17	1	31	152	106	89	183	150

**(b):** 

Number	Fat (%)	BMI (kg/m^2^)	Visceral index	NC (cm)	WC (cm)	HC (cm)	AN score, neck	AN score, axilla
1	35.5	39.7	24	45.5	120.5	131.2	4	4
2	30.5	28	8	36	90.3	99.5	4	4
3	34.4	28.4	9	39.7	98	96	3	2
4	38.7	34.7	16	42	106.5	119.2	4	4
5	25	31.3	16	41.5	100.2	105	4	4
6	34.2	31.9	10	38.5	98.5	103	4	4
7	42.5	36.6	21	38	110	117	4	4
8	37.7	40.5	30	48.5	130	121	4	4
9	41.9	36.7	19	39	115	109	2	3
10	34.4	38.5	25	45	129	116	4	4
11	40.7	33.1	13	37	108	118	4	4
12	43.6	43.4	27	36	108	132	4	4
13	41	34.5	15	43	105	120	2	2
14	43.2	38.7	22	41.5	115	104	3	3
15	38.9	33	12	35	105	116	3	4
16	29.1	33.8	14	38	110	117	2	3
17	41.2	44.8	15	48	146	128	2	3
